# Role of lipoic acid in multiple sclerosis

**DOI:** 10.1111/cns.13793

**Published:** 2021-12-28

**Authors:** Hongsheng Xie, Xiufang Yang, Yuan Cao, Xipeng Long, Huifang Shang, Zhiyun Jia

**Affiliations:** ^1^ Department of Nuclear Medicine West China Hospital Sichuan University Chengdu China; ^2^ Department of Radiology Huaxi MR Research Center (HMRRC) West China Hospital of Sichuan University Chengdu China; ^3^ Mental Health Center West China Hospital Sichuan University Chengdu China; ^4^ Department of Neurology West China Hospital Sichuan University Chengdu China

**Keywords:** efficacy and safety, experimental autoimmune encephalomyelitis, lipoic acid, multiple sclerosis

## Abstract

Lipoic acid (LA) is an endogenous antioxidant that exists widely in nature. Supplementation with LA is a promising approach to improve the outcomes of patients with multiple sclerosis (MS). This systematic review aimed to provide a comprehensive overview of both in vitro and in vivo studies describing the pharmacokinetics, efficacy, safety, and mechanism of LA in MS‐related experiments and clinical trials. A total of 516 records were identified by searching five databases, including PubMed, Web of Science, Embase, Scopus, and Cochrane Library. Overall, we included 20 studies reporting LA effects in cell and mouse models of MS and 12 studies reporting LA effects in patients with MS. Briefly, cell experiments revealed that LA protected neurons by inhibiting the expression of inflammatory mediators and activities of immune cells. Experimental autoimmune encephalomyelitis mouse experiments demonstrated that LA consistently reduced the number of infiltrating immune cells in the central nervous system and decreased the clinical disability scores. Patients with MS showed relatively stable Expanded Disability Status Scale scores and better walking performance with few adverse events after the oral administration of LA. Notably, heterogeneity of this evidence existed among modeling methods, LA usage, MS stage, and trial duration. In conclusion, this review provides evidence for the anti‐inflammatory and antioxidative effects of LA in both in vitro and in vivo experiments; therefore, patients with MS may benefit from LA administration. Whether LA can be a routine supplementary therapy warrants further study.

## Graphical abstract

Lipoic acid protects the central nervous system by immunomodulation and antioxidation. In the periphery, LA prevents inflammatory cells from crossing the BBB and protects brain endothelial cells. In the CNS, LA inhibits the activity of T cells/microglia, decreases the expression of TNF‐α and IFN‐γ, and neutralizes ROS and NO.

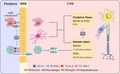

## 1 INTRODUCTION

Multiple sclerosis (MS) is a disabling autoimmune disease of the central nervous system (CNS) characterized by demyelination and neurodegeneration.^1^ It affects approximately 2.5 million people worldwide and poses a growing burden to society.^2^, ^3^ Relapsing‐remitting MS (RRMS) is the most common initial course featuring alternate relapse and remission, and disability is aggravated gradually with illness development.^4^ After approximately 20 years, around 90% of RRMS patients will develop secondary progressive MS (SPMS) characterized by progressive neurodegeneration without any definite remission periods.^5^, ^6^ In addition to SPMS, progressive MS (PMS) also includes primary progressive MS featuring inapparent clinical relapses from the onset. At present, both immune and nonimmune mechanisms are believed to be involved in MS pathogenesis.

The “outside‐in” hypothesis proposes that the inflammatory demyelinating process begins in the subarachnoid space and cortex and extends into the white matter.^7^, ^8^ In this model, the invasion of peripheral immune cells disrupts the blood‐brain barrier (BBB) integrity and contributes to the prolonged presence of inflammatory activity. In RRMS, the interaction of monocytes and brain endothelial cells (ECs) produces massive reactive oxygen species (ROS), leading to the loss of tight junctions and migration of monocytes.^9^ For T cells, the mutual recognition of lymphocyte function‐associated antigen‐1 (LFA‐1), intercellular cell adhesion molecule‐1 (ICAM‐1), very late antigen‐4 (VLA‐4), and vascular cell adhesion molecule‐1 (VCAM‐1) permits them to cross the BBB. The release of matrix metalloprotein‐9 (MMP‐9) by T cells is also essential for the migration process. Notably, infiltrated T cells can recruit macrophages, microglia, and astrocytes by secreting mediators, including tumor necrosis factor‐α (TNF‐α), interferon‐γ (IFN‐γ), and interleukins‐17 (IL‐17).^10^, ^11^ These abnormally activated immune cells target neurons and the myelin sheath and drive MS relapse and progression. Therefore, several disease‐modifying therapies (DMTs) can decrease relapse rates by immunomodulation. However, the potential risks of serious adverse events (AEs) and COVID‐19 infection limit its clinical use to some extent.^12^, ^13^ In PMS, inflammation is compartmentalized and mainly driven by the activities of innate microglia, astrocytes, and B cells.^11^ Unfortunately, the efficacy of DMTs for PMS tends to be disappointing, motivating the search for a new treatment option.^14^

Oxidative stress is another crucial driver of MS once the autoimmune system has caused damage to the CNS.^10^ It occurs when an imbalance exists between excessive production of free radicals and insufficient biological ability to remove them.^15^ The CNS is quite sensitive and vulnerable to oxidative stress because of its high oxygen consumption and lipid abundance. Oxidizing substances, such as ROS and nitrogen species, are usually produced by activated macrophage and microglial structures, causing damage to lipids, proteins, and DNA. Consequently, the CNS is variously disrupted through processes such as increased BBB permeability, myelin phagocytosis, and neurodegeneration.^16^, ^17^ In the plasma of MS patients, the levels of antioxidants and total antioxidant capacity are decreased.^18^, ^19^ Autopsy studies have also widely detected the damage induced by oxidative stress in cerebrospinal fluid and CNS tissues.^20^, ^21^ Therefore, oxidative stress may be another hopeful therapeutic target of MS. At present, many antioxidant compounds have improved serological indicators in MS patients.^22^ Vitamin D decreased the relapse rates as an antioxidant in RRMS patients.^23^ However, the findings of the efficacy of antioxidants tend to be conflicting and confusing, strongly suggesting that the effect of using a single antioxidant is limited. Considering the above, an ideally effective medicine must possess the ability to prevent multiple pathogenic factors and outstanding BBB permeability.

Lipoic acid (LA), also known as thioctic acid, has become a hopeful complementary therapy in MS to target both inflammation and oxidative stress. LA is a double‐sulfhydryl natural antioxidant with two enantiomers according to optical rotation: R‐LA and S‐LA. Overall, R‐LA exists widely in plants and animals, whereas S‐LA is artificially synthesized to compose the racemic mixture (1:1 R/S‐LA).^24^ In the human body, R‐LA is synthesized de novo by cysteine and fatty acids in small amounts; thus, it primarily depends on exogenous supplements such as organ meat, broccoli, and fruits.^25^ For individuals, the racemic form can be absorbed rapidly after oral administration and participate in various biological metabolic pathways. First, it contributes to the synthesis of vitamin C and vitamin E.^26^ Second, it is reduced to dihydro‐LA (DHLA), and DHLA is involved in the biosynthesis of intracellular glutathione (GSH) and coenzyme Q10.^27^, ^28^ Third, R‐LA plays a crucial role in mitochondrial energy production as a cofactor for some enzymatic complexes in the Krebs cycle.^29^ When other metabolic pathways are saturated, redundant LA (nearly 10%) will be excreted through the kidneys.^30^ Over the past two decades, whether LA improves the quality of life of patients with MS has been intensively studied.^31^ In mouse models of experimental autoimmune encephalomyelitis (EAE), LA increased the population of mature oligodendrocytes and alleviated neurological symptoms, suggesting that LA might protect and promote neuronal regeneration.^32^, ^33^ However, the results of alleviated neurological symptoms were inconsistent for different administration pathways, timing, and dosage, making the evidence somewhat fragile. In patients with MS, LA reduced the Expanded Disability Status Scale (EDSS), although the between‐group difference was not statistically significant.^34^, ^35^ The confusing result regarding whether LA could improve patient outcomes probably resulted from the short trial duration. Additionally, the annualized percent brain volume change was less after 2 years of supplementation in the LA group, indicating that LA might prevent neuronal death and reduction.^36^ More importantly, few AEs were reported when using LA as an oral preparation for 2 years. In summary, LA shows strong antioxidative and anti‐inflammatory effects in MS, which makes it a potential candidate for complementary and long‐term therapy.

To date, no study has systematically summarized the current findings of LA in MS, and some results appear to be controversial. A good review of both achievements and limitations will contribute to determining reliable evidence and research trends for future studies. In this review, we aimed to provide comprehensive insight into the role of LA in MS, including the aspects of pharmacokinetics, efficacy, safety, and mechanism, in both in vitro and in vivo experiments. We hope that our work will contribute to the development of new drugs and combination therapy for patients with MS.

## 2 METHODS

### 2.1 Search strategy

According to the guidelines of the 2009 Preferred Reporting Items for Systematic Reviews and Meta‐Analysis (PRISMA) statement,^37^ English‐language studies published from inception up to July 1, 2021, were collected by searching five databases: PubMed (Medline), EMBASE, Web of Science, Scopus, and Cochrane Library. Identified search terms included (“multiple sclerosis” OR “MS”) AND (“lipoic acid” OR “Thioctacid” OR “LA”). Additional records were identified manually through other sources, such as any related review papers and reference lists of all included studies to avoid missing relevant studies in the initial search. The whole search process was conducted by two authors independently (H.S.X. & X.F.Y.).

### 2.2 Study selection

After removing duplicates, all the studies were screened for eligibility by two independent authors (H.S.X. & X.F.Y.). The inclusion criteria in this systematic review included the following: (1) randomized intervention study in patients meeting the McDonald criteria for MS^38^, ^39^; (2) preclinical experiments based on the mouse and cell models of MS; and (3) publications in peer‐reviewed journals. The exclusion criteria included the following: (1) combined other antioxidants; (2) irrelevant endpoints such as biochemical metabolism and visual changes; and (3) nonoriginal studies. All the included studies were cross‐checked, and in‐depth discussions were required to resolve disagreements and make the ultimate decision with the senior author (Z.Y.J.).

### 2.3 Data extraction

For the included studies, we collected the following information into a spreadsheet in Excel: (1) subject characteristics including age, sex, EDSS score, and MS duration; (2) MS‐related model establishment in the preclinical experiments; (3) LA dosage (4) endpoints including efficacy, safety, pharmacokinetics, and mechanism; and (5) first author's name, publication date, study design, and follow‐up duration. For detailed data not shown in the full text, the e‐mails were sent to the corresponding authors for help.

## 3 DISCUSSION

**FIGURE 1:**
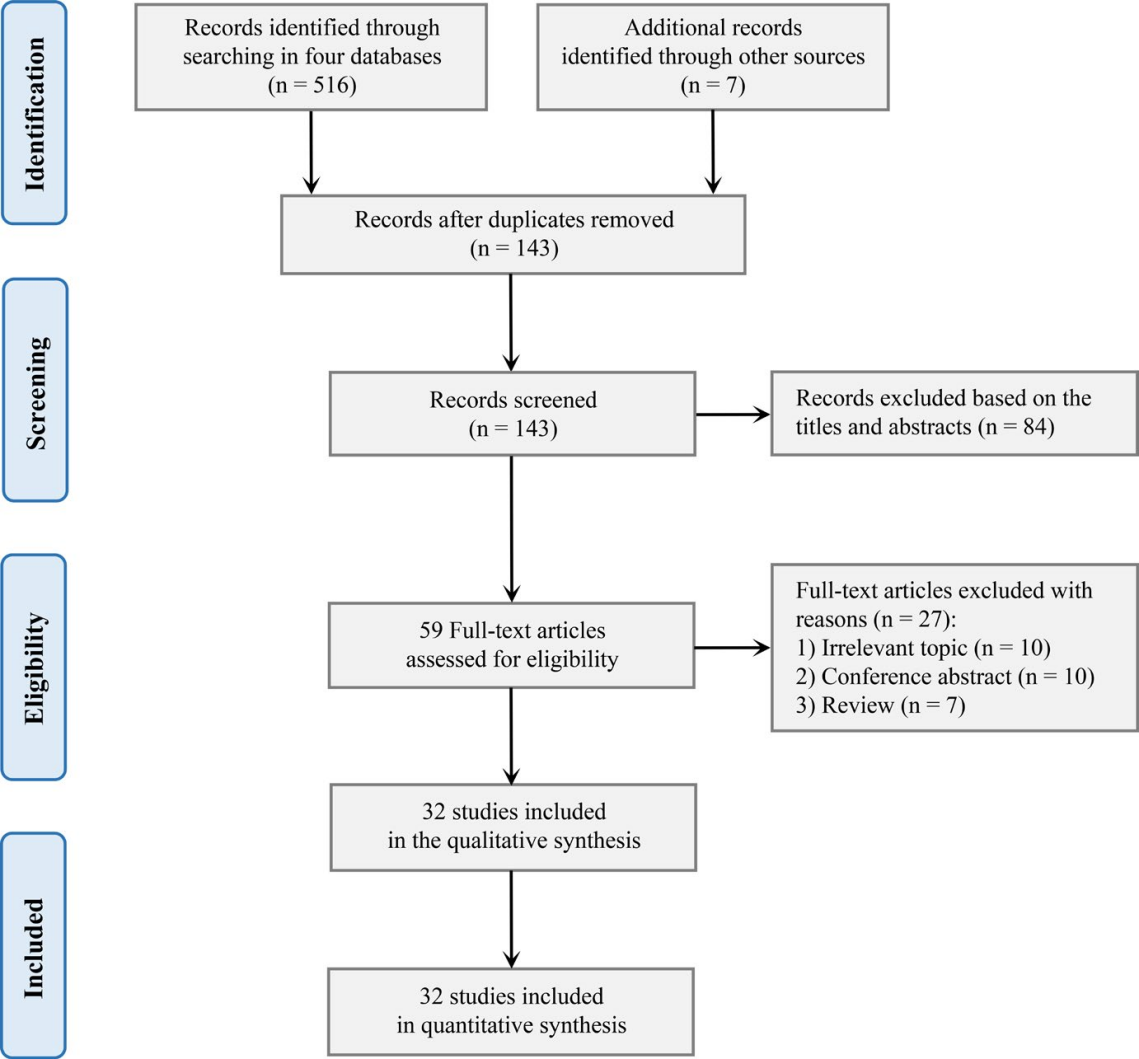
Preferred Reporting Items for Systematic Reviews flowchart

We obtained 516 potential records in the initial systematic search. After the removal of duplicates, 143 studies were screened based on the title and abstract, leading to 59 full‐text studies screened for eligibility. In this process, 27 articles were excluded because of irrelevant endpoints, nonoriginal studies, and combined antioxidant supplements. Finally, 32 intervention studies were included in this systematic review to investigate the effects of LA on efficacy, safety, pharmacokinetics, and mechanism. An overview of the study selection is presented in Figure 1.

### 3.1 LA pharmacokinetics and transportation to the brain

A rat experiment found that the duodenum was the best portion of the intestine for LA absorption and that R‐LA showed a higher absorption percentage than S‐LA.^40^ Notably, two vital pathways are involved in the process of LA crossing the intestinal barrier: Na+/multivitamin (SMVT) and monocarboxylic acid (MCT) transporters.^41^, ^42^ Under equilibrium conditions, human SMVT can simultaneously bind and transport two LA molecules into the mesenteric vein, and human MCT transports LA in an energy‐ and low‐pH‐dependent manner. In the patients with RRMS/SPMS and healthy volunteers, the pharmacokinetic parameters showed no significant difference, suggesting that the MS status did not influence LA metabolism.^43^ In 54 patients with SPMS, pharmacokinetics showed no significant difference between the baseline and 1 year later, suggesting that the oral administration of LA was stable for long‐term use.^44^ In patients with MS, three studies found that the time to reach the peak concentration of R‐LA was much shorter than that of the racemic form, indicating the quicker absorption of the R‐configuration.^33^, ^45^ Additionally, R‐LA showed a much larger area under the curve than the racemic form under the same dosage, indicating the better utilization of the R‐configuration. In summary, R‐LA showed quicker and better utilization than the racemic form, emphasizing the necessity of unified formulations and encapsulations if considering it as a supplementary therapy.

Notably, human SMVT and MCT are also expressed in brain microvessels and contribute to the transportation of LA across the BBB.^46^, ^47^ In an in vitro experiment, LA showed the ability to cross the BBB and exert beneficial effects on the viability of astrocytes.^48^ Besides, a rat experiment found that^14^C‐labeled LA reached peak levels in the cortex, spinal cord, and sciatic nerve after one‐half hour of oral administration, indicating that LA was taken up by both the CNS and peripheral nerves.^49^ LA was also measured in the rat brain cortex, cerebellum, striatum, and hippocampus after intravenous and intraperitoneal administration.^50^, ^51^ Notably, a recent rat experiment found that the LA did not cross the BBB as easily as supposed after the correction for blood volume, which emphasized that the permeability of the BBB might be greatly influenced by cerebral blood flow.^52^

### 3.2 Role of LA in cell experiments

Human peripheral blood mononuclear cells (PBMCs) are isolated from peripheral blood and feature round nuclei. They mainly comprise lymphocytes, monocytes, and NK cells.^53^ Most PBMCs are naïve without immune effects. Importantly, the largest fraction, T cells, will develop into diverse subsets of Th1, Th2, Th17, or regulatory T cells (Treg cells) after activation by different cytokines.^54^, ^55^ Monocytes in PBMCs can also be activated by proinflammatory factors to simulate the immune status of MS. These make human PBMCs a suitable model of MS and provide an opportunity to mirror the autoimmune response in the CNS. Additionally, murine cell models of MS are established directly by isolating and culturing brain cells, including primary microglial cells stimulated with lipopolysaccharide/IFN‐γ and primary cortical neurons treated with H_2_O_2_. We included nine studies based on human PBMCs or murine cells, which were treated with 10–100 μg/ml LA (Table 1). No study reported the specific form of LA, and only one study indicated the usage of both LA and DHLA.

**TABLE 1:** Effects of LA on MS in the preclinical studies

Study	Subjects	LA dosage	Antioxidation	Immunomodulation	Neuroprotection	Duration
Sanadgol et al^32^	36 mice	20–40 mg/kg LA, ip	ROS (–)	NA	OLG (+) Bax/Bcl−2, caspase−3 (–)	5 weeks
Yadav et al^33^	49 mice	5–100 mg/kg R/S LA, ih	NA	NA	10‐Day CDS (–)	7 weeks
Marracci et al^56^	Human T‐cell	50–100 μg/ml LA 25–100 μg/ml DHLA	NA	T‐cell migration (–) VLA−4, MMP−9 (–)	NA	NA
Salinthone et al. 2010^57^	Human PBMC	50–100 μg/ml LA	NA	T‐cell proliferation (–) IL−6, IL−17 (–)	cAMP (+)	NA
Lee et al^58^	Human monocyte	250 mmol/l LA	NF‐κβ (–)	ICAM−1 (–)	NA	NA
George et al^59^	Human PBMC	100 μg/ml LA	NA	Monocyte migration (–) B‐cell migration (–)	NA	NA
Salinthone et al^60^	Human PBMC	10–100 μg/ml LA	NA	NK‐cell cytotoxicity (–) INF‐γ (–)	NA	NA
Fiedler et al^61^	Human PBMC	25–100 μg/ml LA	NA	Phagocytosis (–) IL−1β (–), cAMP (+) TNF‐α, IL−6 (=)	NA	NA
Schillace et al^62^	Human PBMC	100 μg/ml LA	NA	NA	cAMP (+)	NA
Chaudhary et al^63^	Murine microglia	25–100 μg/ml LA	NA	Phagocytosis (–)	NA	NA
Barsukova et al^64^	Murine neurons	100 μg/ml LA	ROS (–)	NA	Axonal integrity, cAMP (+)	NA
Marracci et al^69^	87 mice	10–50 mg/kg R/S LA, sc	NA	T‐cell, MMP−9 (–)	10‐Day CDS (–)	7 weeks
Morini e^70^	45 mice	5 mg/kg LA, orally 50 mg/kg LA, ip	NA	Immune infiltration (–) INF‐γ, IL−4, MMP−9 (–)	Disease scores (–)	6 weeks
Schreibelt et al. 2006 ^71^	14 rats	10–100 mg/kg R/S LA, sc	NA	Monocytes (–)	Clinical signs (–) BBB permeability (–)	3 weeks
Wang et al^72^	20 mice	50 mg/kg LA, injection	PPAR‐γ (+)	Immune infiltration (–) Tregs (+)	Clinical score (–)	3 weeks
Li et al^73^	Mice	100 mg/kg LA, ip	SOD (+) Malondialdehyde (–)	Immune infiltration (–) TNF‐α (–), Tregs (+)	Clinical signs, demyelination (–) Axons (+)	26 weeks
Dietrich et al^74^	Mice	100 mg/kg R/S LA, orally	Glutathione (+)	Immune infiltration (–)	Disability score (–) RGC (+)	17 weeks
Khan et al^75^	24 mice	3–10 mg/kg LA, sc	NA	Immune infiltration (–)	Neuropathic pain (–)	5 weeks
Chaudhary et al^81^	12 mice	100 mg/kg LA, sc	NA	Immune infiltration (–) ICAM−1, VCAM−1 (–)	NA	3 weeks
Chaudhary et al^82^	40 mice	100 mg/kg LA, sc	NA	Immune infiltration (–)	NA	3 weeks

Note

“‐” indicates reduced expression or inhibited activity compared with non‐LA group, and “+” indicates increased expression or enhanced activity.

Abbreviations: BBB, Blood‐brain barrier; cAMP, Cyclic adenosine monophosphate; CDS, Cumulative Disease Score; COX‐2, Cyclooxygenase‐2; ICAM‐1, Intercellular cell adhesion molecule‐1; Ih, Subcutaneous injection; INF‐γ, Interferon‐γ; Ip, Intraperitoneal injection; MMP‐9, Matrix metalloprotein‐9; NA, Not available; OLG, Oligodendrocytes; PBMC, Peripheral blood mononuclear cells; PGE2, Prostaglandin E2; PPAR‐γ, Peroxisome‐proplator‐actified receptor‐γ; RGC, Retinal ganglion cells; ROS, Reactive oxygen species; Sc, Intramuscular injection; SOD, Superoxide dismutase; TNF‐α, Tumor necrosis factor‐α; VCAM‐1, Vascular cell adhesion molecule‐1; VLA‐4, Very late antigen‐4.

Overall, LA inhibited the expression of various inflammatory mediators and the activities of immune cells in human PBMCs. In human T cells, LA inhibited cellular transmigration across a fibronectin barrier in a dose‐dependent manner, likely because LA could downregulate the surface expression of VLA‐4 and decrease the MMP‐9 content in culture supernatants.^56^ Additionally, the oral administration of LA inhibited T‐cell proliferation and activation enriched from the PBMCs of MS patients, which might be related to elevated intracellular cyclic adenosine monophosphate (cAMP).^57^ Further investigation demonstrated a lower content of IL‐6 and IL‐17 in culture supernatants than that in the non‐LA group. In human monocytes, LA inhibited cellular migration in a dose‐dependent manner.^58^, ^59^ Importantly, this effect might be related to the reduced activity of nuclear transcription factor‐kappa B (NF‐KB), leading to the decreased expression of TNF‐α, MMP‐9, and ICAM‐1.^31^ LA also lowered the percentage of phagocytic cells in a dose‐dependent manner in monocytes from both healthy controls and patients with RRMS. In human PBMCs, LA decreased the expression of various proinflammatory cytokines, including IL‐1β, IL‐6, IL‐17, and IFN‐γ.^60^, ^61^ However, some studies reported no difference in expression of TNF‐α and IL‐1β between the LA and non‐LA groups, which may be explained by the modeling approach of lipopolysaccharides and the relatively small sample size. Notably, three studies revealed that the above anti‐inflammatory and neuroprotective effects might be closely associated with elevated intracellular cAMP expression.^62^ Notably, the above outcomes based on PBMCs should be interpreted carefully because they lack in vivo environmental stimuli.

In murine cell models of MS, LA protected neurons and disturbed the activities of immune cells. In murine IFN‐γ‐activated microglia, LA disorganized the actin protein and disturbed the formation of membrane blebs, likely leading to alterations in cellular mobility and phagocytosis.^63^ In murine H_2_O_2_‐treated cortical neurons, oxidative stress led to a marked increase in axoplasmic Ca^2+^ and the formation of the axonal spheroid, where axonal severing occurred. Interestingly, pretreatment with LA completely prevented spheroid formation and maintained axonal integrity by increasing the levels of cAMP.^64^ Presently, the main limitation of cell experiments resides in the nonunified methods of establishing the MS model. CNS cells of EAE mice are likely the most appropriate and convincing model for the double hit of autoimmunity and oxidative stress.

### 3.3 Role of LA in animal experiments

EAE is a reliable murine model that can well simulate the occurrence and development of MS.^65^ In the 11 included studies, EAE induction was accomplished using three methods (Table 1). Five studies indicated that the mice were immunized according to the standard protocol with complete Freund's adjuvant (CFA) containing oligodendrocyte glycoprotein fragment 35–55 or guinea pig myelin basic protein. Four studies indicated that the mice were immunized using CFA containing proteolipid protein (PLP) 139–151 peptide. PLP is a hydrophobic integral membrane protein accounting for half of the protein content of CNS myelin and was recently proven to correlate with the severity of disease in MS patients.^66^ In addition to the autoimmune component, the oral administration of cuprizone can cause whole‐brain demyelination and gliosis and was used to establish the murine model in one study. Compared with the two previous methods, cuprizone induction is easier to operate but time‐consuming (5 *vs*. 2 weeks). More importantly, female mice are more resistant to cuprizone induction, and estradiol/progesterone can protect against cuprizone‐induced demyelination.^67^, ^68^ The LA dosage was 5–100 mg/kg per day, and the mode of administration included intramuscular injection (*n* = 5), intraperitoneal injection (*n* = 3), subcutaneous injection (*n* = 1), oral administration (*n* = 1), and general injection (*n* = 1). Only three studies indicated that a racemic form of LA was used, and the remaining eight studies did not report the specific form. The range of the experiment duration was from 3 to 26 weeks. Notably, by comparing the serum pharmacokinetic parameters, a 50 mg/kg subcutaneous dose in symptom‐remitted mice was considered equal to a dose of 1200 mg of LA in patients with MS.^33^

#### 3.3.1 LA efficacy in EAE mice

Overall, seven studies revealed that LA reduced the MS‐related neurological scores focusing on the weak/spastic tail and limb paralysis, and symptomatic relief emerged approximately one week after the onset.^33^, ^69^, ^70^, ^71^, ^72^, ^73^, ^74^ Notably, three studies indicated that the improvement was dose‐dependent.^33^, ^69^, ^70^ More importantly, two studies pointed out that only preventive usage of LA could alleviate the clinical signs, whereas the oral supplement after the onset did not function well in mice.^70^, ^74^ One possible explanation might be the low dose of 5 mg/kg orally. Therefore, demonstrating the efficacy of oral administration should be highlighted in future as the target mode in MS patients. Additionally, LA was also effective in alleviating MS‐associated neuropathic pain.^75^ Consistent with our results, a systematic review evaluated LA as effective in improving demyelination and neurobehaviors in large animal numbers.^76^ In summary, EAE mouse experiments demonstrated that LA effectively improved neurological outcomes when the injection was at relatively high doses.

#### 3.3.2 LA mechanisms in EAE mice

**FIGURE 2:**
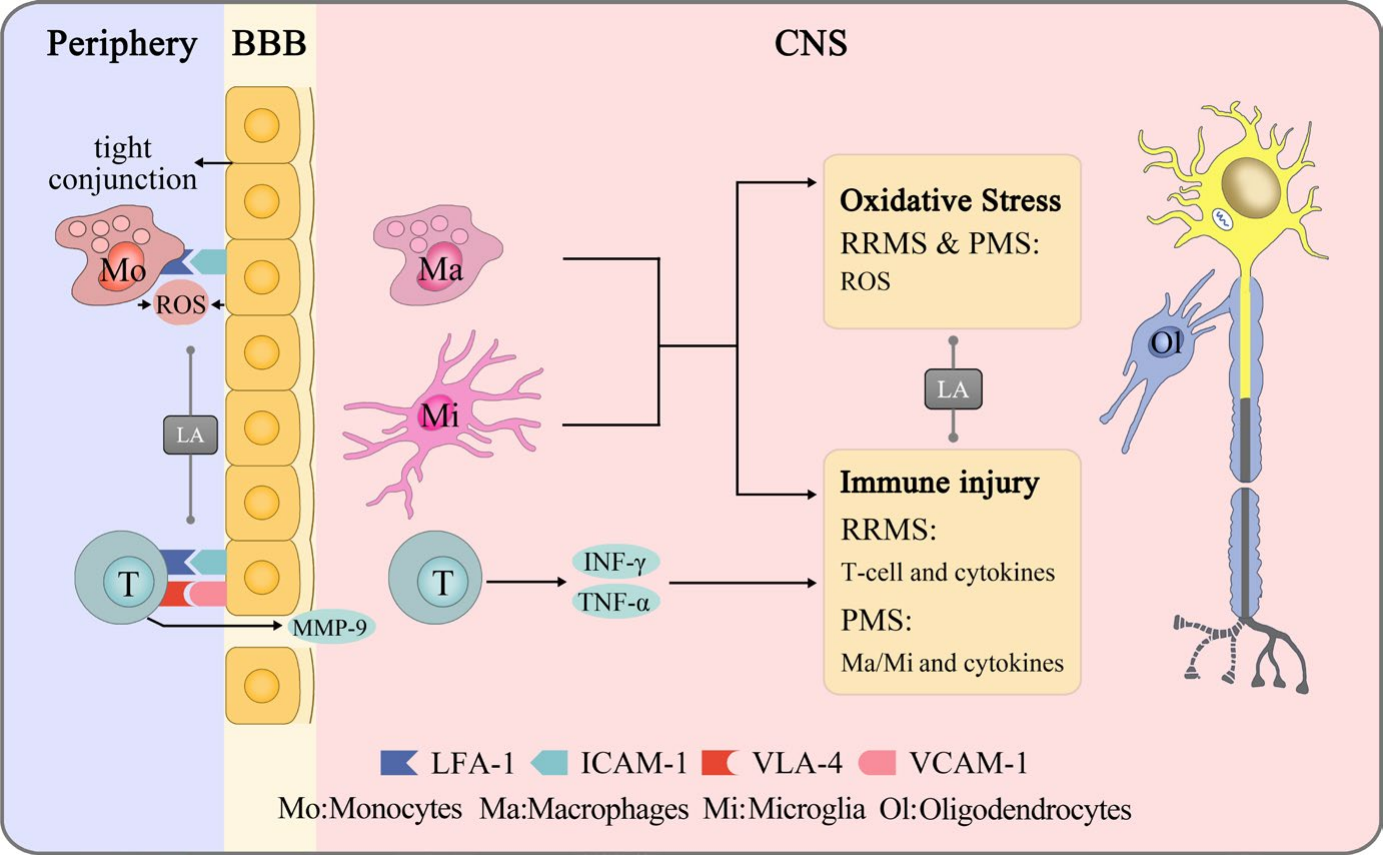
Lipoic acid protects the central nervous system by immunomodulation and antioxidation. In the periphery, LA prevents inflammatory cells from crossing the BBB by inhibiting the expression of LFA‐1, ICAM‐1, VLA‐4, VCAM‐1, and MMP‐9 and protects brain endothelial cells. In the CNS, LA modulates autoimmunity by inhibiting the activity of T cells/microglia and decreasing the expression of TNF‐α and IFN‐γ, and LA reduces oxidative stress by neutralizing ROS and NO

Nuclear factor erythroid‐2 related factor 2 (Nrf2) is a redox‐sensitive transcription factor existing in the cytoplasm, and it will translocate to the nucleus when oxidative stress occurs.^77^ In the nucleus, it binds to antioxidant response elements and initiates the transcription of over 200 detoxification genes.^78^ In the rat brain, LA promoted Nrf2 translocation and the superoxide dismutase (SOD) activity to defend against oxidative stress.^79^ Besides, LA upregulated the expression of Nrf2 and its downstream hemeoxygenase‐1 to alleviate neuronal cell apoptosis.^80^ In EAE mice, LA increased the expression of GSH and SOD to enhance antioxidant system activity (Figure 2). Meanwhile, LA decreased the levels of ROS and lipid peroxidation in EAE mice.^32^ These findings suggested that the LA‐Nrf2‐antioxidative system pathway might be involved in neurological improvement in EAE mice.

LA inhibited the activity of immune cells and reduced inflammatory infiltration in the CNS. In terms of pathological evidence, five studies found that LA reduced CD3+/CD4+ T‐cell infiltration in the brain and spinal cord, and three studies indicated reduced macrophage/microglial infiltration in EAE mice.^81^, ^82^ Above all, LA downregulated the expression of ICAM‐1 and VCAM‐1 in brain endothelial cells by colocalization analysis, contributing to the impairment of peripheral immune cell migration.^81^ For T cells, two studies revealed that LA increased Treg cell levels and decreased encephalitogenic T‐cell levels, leading to a lower grade of the inflammatory response.^72^, ^73^ LA also inhibited T‐cell activities by reducing the expression of MMP‐9 in a dose‐dependent manner to protect BBB integrity.^69^, ^70^ Furthermore, LA downregulated CD4 from the surface of Jurkat cells in a concentration‐dependent manner. Interestingly, CD4 inhibitors reduced the severity of EAE symptoms in mice.^83^ These findings indicated that LA could effectively decrease the invasion of peripheral immune cells and the inflammatory response in the CNS.

NF‐KB is a classical proinflammatory transcription factor existing in the cytoplasm, and it translocates to the nucleus and promotes the transcription of TNF‐α and IL‐6 during the oxidative stress.^84^ On the other hand, LA could directly inhibit the activity of NF‐KB and its downstream protein expression.^85^ On the contrary, the enhanced antioxidative capability promoted by Nrf2 would also suppress the NF‐KB activity. Besides, Nrf2 can inhibit the transcription of the various inflammatory mediators, including TNF‐α and IL‐6 in microglia and astrocytes.^86^, ^87^ In EAE mice, two studies indicated that LA decreased the expression of MMP‐9 to protect the BBB from immune disruption.^69^, ^70^ Additionally, LA also decreased the expression of TNF‐α, IFN‐γ, and IL‐4 to reduce the level of inflammation in EAE mice.^73^ In future, it is vital to further explore the role of NF‐KB and Nrf2 in the anti‐inflammatory effects of LA, and thus develop new potential therapeutic targets for MS.

Peroxisome proliferator‐activated receptor‐γ (PPAR‐γ) is a ligand‐activated transcription factor that widely exists in neurons, astrocytes, and microglia.^88^ PPAR‐γ can promote the expression of catalase and SOD to enhance the antioxidative capability and decrease the NF‐KB expression to alleviate the immune response.^89^, ^90^ In EAE mice, LA induced the activation of both endogenous and central PPAR‐γ and reduced inflammatory injury.^72^ More importantly, PPAR‐γ can protect neurons from apoptosis by regulating the expression of B‐cell lymphoma‐2 (Bcl‐2) and Bcl‐2‐associated X protein (Bax).^91^ In EAE mice, LA increased the population of mature oligodendrocytes and decreased the expression of caspase‐3 and the Bax/Bcl‐2 ratio, indicating that LA might contribute to maintaining the integrity of axons.^32^ Additionally, LA contributed to the higher viability of retinal ganglion cells and increased myelin basic protein expression after over four months of administration.^73^, ^74^ These findings suggested that the LA‐PPAR‐γ‐neuroprotection pathway might be involved in neurological improvement in EAE mice.

### 3.4 Role of LA in clinical trials

Twelve studies based on clinical trials were included to describe the role of LA in patients with MS (Table 2). A total of 410 patients were included: 38% with RRMS, 38% with SPMS, 4% with PMS, and 20% with MS. The average illness duration was 4–30 years. The LA dosage was 600–2400 mg/day orally, and seven studies indicated the specific form (racemic form = 5; both R‐LA and racemic form = 2). The average trial duration was 2–96 weeks.

**TABLE 2:** Effects of LA on MS in the clinical studies

Study	Patients	Illness duration (years ± SD)	LA dosage (orally/day)	Serious adverse events	Topic	Study design	Trial Duration	Main findings
Yadav et al^33^	12 RRMS 12 PMS	12.5 (1.0–35.0)	1200 mg R/S‐LA	NA	PK	Open label, RCT	NA	1200mg oral lipoic acid can achieve therapeutic serum levels.
Khalili, et al^34^	24 RRMS	5.2 ± 4.9	1200 mg LA	NA	Mechanism Efficacy	Double‐blind, placebo‐controlled, RCT	12 weeks	ADMA (–) EDSS, new enhanced plaque (=)
Khalili, et al^35^	46 RRMS	4.9 ± 3.8	1200 mg LA	NA	Mechanism Efficacy	Double‐blind, placebo‐controlled, RCT	12 weeks	INF‐γ, IL−4, ICAM−1, TGF‐β (–) TNF‐α, IL−6, MMP−9, EDSS (=)
Spain, et al^36^	51 SPMS	29.4 ± 9.6	1200 mg R/S‐LA	2 Gastrointestinal disorders 2 Renal disorders 1 Rash 1 NA	Efficacy Safety	Double‐blind, placebo‐controlled, RCT	96 weeks	Brain atrophy rate (–) T25FW (=) Safe and high compliance
Fiedler et al. 2018 ^43^	21 RRMS 16 SPMS 20 HC	NA	1200 mg R/S‐LA	NA	PK Mechanism	Phase I, open label	NA	PK parameters showed no statistical difference in 3 groups. cAMP: RRMS (–); SPMS, HC (+)
Bittner, et al^44^	54 SPMS	30.9 ± 9.3	1200 mg R/S‐LA	2 Renal disorders 1 Gastrointestinal disorder 1 Testicular cancer	Safety PK	Placebo‐controlled RCT	48 weeks	Fall events (–) Stable PK parameters
Salinthone et al^45^	28 MS	NA	1200 mg R‐LA, R/S‐LA	NA	PK Mechanism	RCT	NA	R‐LA *vs* R/S‐LA: AUC (+), Tmax, cAMP (–)
Loy et al^95^	21 SPMS	25.9 ± 8.9	1200 mg LA	NA	Efficacy	Double‐blind, placebo‐controlled, RCT	96 weeks	Walking performance (+)
NCT03161028 Recruiting, ^97^	118 PMS		1200 mg LA	NA	Efficacy Safety	Phase II placebo‐controlled, RCT	96 weeks	Endpoints: T25FW, fall count, brain atrophy, and adverse events.
Cameron, et al^98^	15 SPMS 5 PMS	26.0 ± 10.3	600 mg R‐LA 1200mg R/S‐LA	None	Safety PK	Double‐blind, cross‐over	3 weeks	R‐LA showed better gastrointestinal tolerability and serum absorption.
Yadav, et al^100^	33 MS	4.0 (0.0–6.5)	1200 mg LA 2400 mg LA	3 Nausea 1 Allergic rash	Safety Mechanism	Double‐blind, placebo‐controlled, RCT	2 weeks	MMP−9, ICAM−1 (–) Well‐tolerated
Khalili, et al^106^	52 RRMS	4.9 ± 3.8	1200 mg R/S‐LA	NA	Mechanism	Double‐blind, placebo‐controlled, RCT	12 weeks	TAC (+) SOD, GPX, MAD (=)
Waslo et al^107^	20 MS	NA	LA	NA	Mechanism Efficacy	Placebo‐controlled	48 weeks	GSH/GSSG ratio (=)

Note

“‐” indicates decreased expression or event compared with non‐LA group, “+” indicates increased expression or enhanced activity, and “=” indicates no statistical difference.

Abbreviations: ADMA, asymmetric dimethylarginine; cAMP, cyclic adenosine monophosphate; EDSS, Expanded Disability Status Scale; GPX, glutathione peroxidase; HC, healthy control; ICAM‐1, intercellular cell adhesion molecule‐1; IL‐, interleukin‐; INF‐γ, interferon‐γ; MAD, malondialdehyde; MMP‐9, matrix metalloprotein‐9; NA, not available; PK, pharmacokinetics; SOD, superoxide dismutase; T25FW, Timed 25‐Foot Walk; TAC, total antioxidant capacity; TGF‐β, transforming growth factor‐β; TNF‐α, tumor necrosis factor‐α.

#### 3.4.1 LA efficacy and safety

EDSS is the most common method to evaluate the disability severity of patients with MS and has recently been proven to positively correlate with the mean volume of T1 hypointense lesions.^92^ Specifically, the EDSS score in the LA group was stable or slightly reduced after 12 weeks, whereas that in the placebo group was slightly increased.^34^, ^35^ Additionally, both baseline EDSS scores in the LA cohort were higher than those in the placebo group. Thus, the EDSS improvement by LA might be insufficient to compensate for the initial difference. Similarly, the treatment effects of DMTs were reported to be dramatically influenced by the baseline EDSS scores.^93^ Therefore, positive consequences can result if the follow‐up duration is longer. Another study revealed a dramatic decrease in the EDSS score after oral administration for at least 48 weeks; the improvement in the EDSS score was likely correlated with the lowering of soluble ICAM‐1.^94^ For SPMS patients, 71% of subjects in the placebo cohort showed stable or improved EDSS scores, and 61% of patients in the LA cohort appeared the same after the 96 weeks follow‐up.^36^ Thus, exploring the long‐term effect on the EDSS score is meaningful and necessary. In addition to the EDSS score, LA contributed to a 68% reduction in the annualized percent change in brain volume in SPMS subjects after a 2 years follow‐up.^36^ Additionally, Spain reported that LA improved the Timed 25‐Foot Walk and reduced falling events in SPMS patients. Similar improvements in walking performance of SPMS subjects treated with LA were also revealed by another 2 years, double‐blind, placebo‐controlled RCT.^95^ Notably, 39 MS patients in the LA group showed fewer new enhanced plaques in the magnetic resonance imaging exam, indicating its potential antirelapse effects.^96^ Presently, a multicenter placebo‐controlled RCT is recruiting patients and sets walking performance as the primary endpoint with comprehensive clinical evaluations.^97^ In summary, pilot studies have demonstrated the efficacy of LA though the evidence is imitated.

LA appears quite safe, with a compliance rate of 80% to 97% in MS patients.^44^ The most common adverse events are gastrointestinal intolerance and rash. Notably, the oral administration of 600 mg of R‐LA showed approximately half less gastrointestinal discomfort than 1200 mg of the racemic form, while the bioavailability was nearly equivalent.^98^ Taking LA after meals and enteric coating can also improve tolerability.^99^ Furthermore, one case of maculopapular rash with fever was reported after 2400 mg/day for 1 week, and the symptoms were resolved only by stopping intake.^100^ The other three cases of rash were found in two double‐blind RCTs, but they seemed to be milder and only affected the skin.^36^, ^101^ Consistently, in patients with diabetic polyneuropathy or other CNS diseases, few AEs were found, and some studies did not set AEs as an endpoint.^102^, ^103^, ^104^ Consistent with our results, a meta‐analysis including 71 placebo‐controlled clinical studies found that LA was associated with no increased risk of AEs, even with pregnancy status.^105^ For future studies, seeking the balance of effective dose and fewer AEs will be necessary.

#### 3.4.2 LA mechanisms in MS patients

LA showed mixed antioxidative and anti‐inflammatory effects (Figure 2). A 12 weeks double‐blind RCT reported an apparent improvement in serum total antioxidant capacity in 52 RRMS subjects.^106^ However, the specific approach remained confusing because no difference was found in the serum GSH:GSSG ratio, superoxide dismutase, and GSH peroxidase activity.^34^, ^107^ Notably, LA reduced the content of asymmetric dimethylarginine (a major endogenous inhibitor of endothelial NO synthase) in the blood of MS patients.^108^ In future, considering that LA can chelate heavy metals, including iron and copper, investigating whether LA can function to prevent gadolinium‐related contrast magnetic resonance imaging (MRI) will be interesting.^109^ Additionally, another key point is whether improved peripheral antioxidant capacity can exactly reflect the redox status in the CNS, a topic that warrants investigation. In summary, LA is one of the most promising antioxidants to alleviate oxidative stress in the CNS because of its high water and fat solubility.

Regarding immunomodulation, LA protects the BBB from disruption by peripheral inflammatory cells. On the one hand, MMP‐9 released by T cells degrades components of the extracellular matrix,^110^ and ROS produced during monocyte binding to ECs result in the loss of tight junctions.^9^ On the contrary, when endothelial cells are activated by TNF‐α/IFN‐γ, ICAM‐1 is overexpressed and binds to LFA‐1, initiating cytoskeletal rearrangement in brain ECs.^111^, ^112^ These events that disrupt the BBB explain the finding that high levels of MMP‐9/ICAM‐1 are present before the appearance of new MRI‐based gadolinium‐enhancing lesions in MS patients.^113^, ^114^, ^115^ In response, a two‐week LA supplement decreased the levels of serum MMP‐9 and ICAM‐1 in 33 patients.^100^ Another twelve weeks of LA supplementation decreased the levels of serum IFN‐γ and IL‐4 in 46 RRMS patients, but the serum TNF‐α and IL‐6 levels showed no difference.^35^ Notably, a recent study found that the oral administration of meglumine cAMP promoted BBB integrity, suggesting that LA may maintain the normal functioning condition of ECs through a similar effect.^116^ In summary, by stabilizing the BBB, LA can disturb inflammation progression in the CNS. Additionally, using LA orally appears to benefit MS patients and help to reduce relapse tendency. Future studies should note the clinical heterogeneity of a relatively short trial duration, LA forms, and different MS stages.

## 4 CONCLUSIONS

We comprehensively summarized the current findings of LA regarding pharmacokinetics, efficacy, safety, and mechanisms in MS while critically proposing deficiencies and improvements for future studies. Overall, LA exerted positive neuroprotective effects by antioxidation and immunomodulation in both in vitro and in vivo experiments. LA decreased the clinical disability scores in EAE mice and halted the worsening of EDSS scores without any serious AEs in MS patients. Notably, R‐LA showed better bioavailability and gastrointestinal tolerance than the same dosage of the racemic form. Because demyelination, oxidative stress, and autoimmunity are typical features of MS, using LA as a dietary supplement or in combination therapy is a hopeful and safe strategy in future. The limitations of this study are that a certain form of LA is not always reported. Although we tried to contact the authors by e‐mail, only one replied to us patiently. Second, the outcomes of mouse experiments may not be generalizable to patients because of the injection administration. Finally, and inevitably, the evidence might be incomplete because of the subjectivity of search terms and combinations.

To date, the achievements of LA supplementation are exciting, but the evidence is not sufficiently strong, being limited primarily by the short trial duration and insufficient study quantity. Thus, multicenter and long‐term controlled studies are encouraged to determine the strength of LA orally, an appropriate dose for long‐term usage, and the most suitable combination therapy. As our understanding of the role of LA improves, we hope to uncover the best treatment regimens for MS patients.

## CONFLICTS OF INTEREST

The authors declare no financial or other conflicts of interest.

## AUTHOR CONTRIBUTIONS

**Hongsheng Xie** contributed to investigation, data curation, and writing (original draft). **Xiufang Yang** contributed to investigation, data curation, and writing (editing). **Yuan Cao** involved in data curation. **Xipeng Long** contributed to validation and resources. **Huifang Shang** involved in investigation. **Zhiyun Jia** contributed to conceptualization, supervision, writing (revising), and funding acquisition.

## Data Availability

Data sharing is not applicable to this article as no new data were created or analyzed in this study.

## References

[1] Filippi M , Bar‐Or A , Piehl F , et al. Multiple sclerosis. Nat Rev Dis Primers. 2018;4(1):43. 10.1038/s41572-018-0041-4 30410033

[2] Oh J , Vidal‐Jordana A , Montalban X . Multiple sclerosis: clinical aspects. Curr Opin Neurol. 2018;31(6):752‐759. 30300239 10.1097/WCO.0000000000000622

[3] Abboud H , Serra A . The pressing questions in multiple sclerosis care in the era of COVID‐19. J Neurol Sci. 2020;416:117005. 32599294 10.1016/j.jns.2020.117005 PMC7308765

[4] Sl H . Progress in multiple sclerosis research: an example of bedside to Bench. JAMA. 2020;324(9):841‐842. 32379266 10.1001/jama.2020.1522 PMC7990471

[5] Rovaris M , Confavreux C , Furlan R , Kappos L , Comi G , Filippi M . Secondary progressive multiple sclerosis: current knowledge and future challenges. Lancet Neurol. 2006;5(4):343‐354. 16545751 10.1016/S1474-4422(06)70410-0

[6] Bross M , Hackett M , Bernitsas E . Approved and emerging disease modifying therapies on neurodegeneration in multiple sclerosis. Int J Mol Sci. 2020;21(12):1‐15. 10.3390/ijms21124312 PMC7348940 32560364

[7] Malpass K . ‘Outside‐in’ demyelination in MS. Nat Rev Neurol. 2012;8(2):61. 22249838 10.1038/nrneurol.2011.217

[8] Lucchinetti CF , Popescu BF , Bunyan RF , et al. Inflammatory cortical demyelination in early multiple sclerosis. N Engl J Med. 2011;365(23):2188‐2197. 22150037 10.1056/NEJMoa1100648 PMC3282172

[9] Van der Goes A , Wouters D , Van Der Pol SM , et al. Reactive oxygen species enhance the migration of monocytes across the blood‐brain barrier in vitro. FASEB J. 2001;15(10):1852‐1854. 11481252 10.1096/fj.00-0881fje

[10] Baecher‐Allan C , Kaskow BJ , Weiner HL . Multiple Sclerosis: mechanisms and Immunotherapy. Neuron. 2018;97(4):742‐768. 29470968 10.1016/j.neuron.2018.01.021

[11] Correale J , Gaitán MI , Ysrraelit MC , Fiol MP . Progressive multiple sclerosis: from pathogenic mechanisms to treatment. Brain. 2017;140(3):527‐546. 27794524 10.1093/brain/aww258

[12] Brück W , Gold R , Lund BT , et al. Therapeutic decisions in multiple sclerosis: moving beyond efficacy. JAMA Neurology. 2013;70(10):1315‐1324. 23921521 10.1001/jamaneurol.2013.3510 PMC4106803

[13] Louapre C , Collongues N , Stankoff B , et al. Clinical characteristics and outcomes in patients with coronavirus disease 2019 and multiple sclerosis. JAMA Neurology. 2020;77(9):1079‐1088. 32589189 10.1001/jamaneurol.2020.2581 PMC7320356

[14] Faissner S , Plemel JR , Gold R , Yong VW . Progressive multiple sclerosis: from pathophysiology to therapeutic strategies. Nat Rev Drug Discovery. 2019;18(12):905‐922. 31399729 10.1038/s41573-019-0035-2

[15] Dadhania VP , Trivedi PP , Vikram A , Tripathi DN . Nutraceuticals against neurodegeneration: a mechanistic insight. Curr Neuropharmacol. 2016;14(6):627‐640. 26725888 10.2174/1570159X14666160104142223 PMC4981739

[16] van Horssen J , Witte ME , Schreibelt G , de Vries HE . Radical changes in multiple sclerosis pathogenesis. Biochem Biophys Acta. 2011;1812(2):141‐150. 20600869 10.1016/j.bbadis.2010.06.011

[17] Fiorini A , Koudriavtseva T , Bucaj E , et al. Involvement of oxidative stress in occurrence of relapses in multiple sclerosis: the spectrum of oxidatively modified serum proteins detected by proteomics and redox proteomics analysis. PLoS One. 2013;8(6):e65184. 23762311 10.1371/journal.pone.0065184 PMC3676399

[18] Besler HT , Çomogˇlu S . Lipoprotein oxidation, plasma total antioxidant capacity and homocysteine level in patients with multiple sclerosis. Nutr Neurosci. 2003;6(3):189‐196. 12793524 10.1080/1028415031000115945

[19] Besler H , Comoğlu S , Okçu Z . Serum levels of antioxidant vitamins and lipid peroxidation in multiple sclerosis. Nutr Neurosci. 2002;5:215‐220. 12041878 10.1080/10284150290029205

[20] Langemann H , Kabiersch A , Newcombe J . Measurement of low‐molecular‐weight antioxidants, uric acid, tyrosine and tryptophan in plaques and white matter from patients with multiple sclerosis. Eur Neurol. 1992;32(5):248‐252. 1521544 10.1159/000116835

[21] Ah C , Pt M , Rm K , Re S , Tp M . Peroxynitrite formation within the central nervous system in active multiple sclerosis. J Neuroimmunol. 1998;88:45‐56. 9688323 10.1016/s0165-5728(98)00078-2

[22] Miller ED , Dziedzic A , Saluk‐Bijak J , Bijak M . A review of various antioxidant compounds and their potential utility as complementary therapy in multiple sclerosis. Nutrients. 2019;11(7):1528. 10.3390/nu11071528 PMC6682972 31284389

[23] Shaygannejad V , Janghorbani M , Ashtari F , Dehghan H . Effects of adjunct low‐dose vitamin d on relapsing‐remitting multiple sclerosis progression: preliminary findings of a randomized placebo‐controlled trial. Mult Scler Int. 2012;2012:452541. 22567287 10.1155/2012/452541 PMC3337486

[24] Salehi B , Berkay Yılmaz Y , Antika G , et al. Insights on the use of α‐lipoic acid for therapeutic purposes. Biomolecules. 2019;9(8):356. 10.3390/biom9080356 PMC6723188 31405030

[25] Tibullo D , Li Volti G , Giallongo C , et al. Biochemical and clinical relevance of alpha lipoic acid: antioxidant and anti‐inflammatory activity, molecular pathways and therapeutic potential. Inflamm Res. 2017;66(11):947‐959. 10.1007/s00011-017-1079-6 28676917

[26] Scholich H , Murphy ME , Sies H . Antioxidant activity of dihydrolipoate against microsomal lipid peroxidation and its dependence on alpha‐tocopherol. Biochem Biophys Acta. 1989;1001(3):256‐261. 2492825 10.1016/0005-2760(89)90108-2

[27] Busse E , Zimmer G , Schopohl B , Kornhuber B . Influence of alpha‐lipoic acid on intracellular glutathione in vitro and in vivo. Arzneimittelforschung. 1992;42(6):829‐831. 1418040

[28] Ve K , Ea S , Gm K , et al. Antioxidant action of ubiquinol homologues with different isoprenoid chain length in biomembranes. Free Radic Biol Med. 1990;9(2):117‐126. 2227528 10.1016/0891-5849(90)90114-x

[29] Packer L , Witt EH , Tritschler HJ . Alpha‐Lipoic acid as a biological antioxidant. Free Radic Biol Med. 1995;19(2):227‐250. 7649494 10.1016/0891-5849(95)00017-r

[30] Teichert J , Hermann R , Ruus P , Preiss R . Plasma kinetics, metabolism, and urinary excretion of alpha‐lipoic acid following oral administration in healthy volunteers. J Clin Pharmacol. 2003;43(11):1257‐1267. 14551180 10.1177/0091270003258654

[31] Seifar F , Khalili M , Khaledyan H , et al. α‐Lipoic acid, functional fatty acid, as a novel therapeutic alternative for central nervous system diseases: a review. Nutr Neurosci. 2019;22(5):306‐316. 29185388 10.1080/1028415X.2017.1386755

[32] Sanadgol N , Golab F , Askari H , Moradi F , Ajdary M , Mehdizadeh M . Alpha‐lipoic acid mitigates toxic‐induced demyelination in the corpus callosum by lessening of oxidative stress and stimulation of polydendrocytes proliferation. Metab Brain Dis. 2018;33(1):27‐37. 29022246 10.1007/s11011-017-0099-9

[33] Yadav V , Marracci GH , Munar MY , et al. Pharmacokinetic study of acid in multiple sclerosis: comparing mice and human pharmacokinetic parameters. Multiple Sclerosis. 2010;16(4):387‐397. 10.1177/1352458509359722 20150394 PMC3489916

[34] Khalili M , Soltani M , Moghadam SA , Dehghan P , Azimi A , Abbaszadeh O . Effect of alpha‐lipoic acid on asymmetric dimethylarginine and disability in multiple sclerosis patients: a randomized clinical trial. Electron Physician. 2017;9(7):4899‐4905. 28894553 10.19082/4899 PMC5587011

[35] Khalili M , Azimi A , Izadi V , et al. Does lipoic acid consumption affect the cytokine profile in multiple sclerosis patients: a double‐blind, placebocontrolled, randomized clinical trial. NeuroImmunoModulation. 2014;21(6):291‐296. 10.1159/000356145 24821457

[36] Spain R , Powers K , Murchison C , et al. Lipoic acid in secondary progressive MS: a randomized controlled pilot trial. Neurol Neuroimmunol Neuroinflamm. 2017;4(5):e374. 28680916 10.1212/NXI.0000000000000374 PMC5489387

[37] Moher D , Liberati A , Tetzlaff J , Altman DG . Preferred reporting items for systematic reviews and meta‐analyses: the PRISMA statement. PLoS Med. 2009;6(7):e1000097. 19621072 10.1371/journal.pmed.1000097 PMC2707599

[38] Polman CH , Reingold SC , Banwell B , et al. Diagnostic criteria for multiple sclerosis: 2010 revisions to the McDonald criteria. Ann Neurol. 2011;69(2):292‐302. 21387374 10.1002/ana.22366 PMC3084507

[39] McDonald WI , Compston A , Edan G , et al. Recommended diagnostic criteria for multiple sclerosis: guidelines from the international panel on the diagnosis of multiple sclerosis. Ann Neurol. 2001;50(1):121‐127. 11456302 10.1002/ana.1032

[40] Amenta F , Buccioni M , Ben DD , et al. Ex‐vivo absorption study of lysine R‐lipoate salt, a new pharmaceutical form of R‐ALA. Eur J Pharm Sci. 2018;118:200‐207. 29597044 10.1016/j.ejps.2018.03.025

[41] Zehnpfennig B , Wiriyasermkul P , Carlson DA , Quick M . Interaction of α‐lipoic acid with the human Na+/multivitamin transporter (hSMVT)*. J Biol Chem. 2015;290(26):16372‐16382. 25971966 10.1074/jbc.M114.622555 PMC4481234

[42] Takaishi N , Yoshida K , Satsu H , Shimizu M . Transepithelial transport of α‐lipoic acid across human intestinal Caco‐2 cell monolayers. J Agric Food Chem. 2007;55(13):5253‐5259. 10.1021/jf063624i 17536819

[43] Fiedler SE , Yadav V , Kerns AR , et al. Lipoic acid stimulates cAMP production in healthy control and secondary progressive MS subjects. Mol Neurobiol. 2018;55(7):6037‐6049. 29143287 10.1007/s12035-017-0813-y PMC5953756

[44] Bittner F , Murchison C , Koop D , Bourdette D , Spain R . Lipoic acid pharmacokinetics at baseline and 1 year in secondary progressive MS. Neurol Neuroimmunol Neuroinflamm. 2017;4(5):e380. 28680918 10.1212/NXI.0000000000000380 PMC5489386

[45] Salinthone S , Yadav V , Ganesh M , et al. Comparing the bioavailability of two forms of lipoic acid in multiple sclerosis. BMC Complement Altern Med. 2012;12.239.23190573

[46] Vatine GD , Al‐Ahmad A , Barriga BK , et al. Modeling Psychomotor retardation using iPSCs from MCT8‐deficient patients indicates a prominent role for the blood‐brain barrier. Cell Stem Cell. 2017;20(6):831‐843. 28526555 10.1016/j.stem.2017.04.002 PMC6659720

[47] Uchida Y , Ito K , Ohtsuki S , Kubo Y , Suzuki T , Terasaki T . Major involvement of Na(+) ‐dependent multivitamin transporter (SLC5A6/SMVT) in uptake of biotin and pantothenic acid by human brain capillary endothelial cells. J Neurochem. 2015;134(1):97‐112. 25809983 10.1111/jnc.13092

[48] Molinari C , Morsanuto V , Ghirlanda S , et al. Role of combined lipoic acid and vitamin D3 on astrocytes as a way to prevent brain ageing by induced oxidative stress and iron accumulation. Oxid Med Cell Longev. 2019;2019:2843121. 30944691 10.1155/2019/2843121 PMC6421749

[49] Borbe HO , Peter G . Distribution of [7,8‐14C] a‐lipoic acid in selected organs of the rat after single oral and intravenous administration with special respect to nervous tissues. Arzneimittleforsch/Drug Res. 1995; (in press).

[50] Panigrahi M , Sadguna Y , Shivakumar BR , et al. α‐Lipoic acid protects against reperfusion injury following cerebral ischemia in rats. Brain Res. 1996;717(1):184‐188. https://www.sciencedirect.com/science/article/pii/0006899396000091 8738270 10.1016/0006-8993(96)00009-1

[51] Arivazhagan P , Panneerselvam SR , Panneerselvam C . Effect of DL‐α‐lipoic acid on the status of lipid peroxidation and lipids in aged rats. J Gerontol A Biol Sci Med Sci. 2003;58(9):B788‐B791. 14528033 10.1093/gerona/58.9.b788

[52] Chng HT , New LS , Neo AH , Goh CW , Browne ER , Chan EC . Distribution study of orally administered lipoic acid in rat brain tissues. Brain Res. 2009;1251:80‐86. 19046949 10.1016/j.brainres.2008.11.025

[53] Kleiveland CR , et al. Peripheral blood mononuclear cells. In Verhoeckx K , Cotter P , López‐Expósito I , (Eds.). The Impact of Food Bioactives on Health: In vitro and ex vivo models. Springer Copyright 2015, The Author(s); 2015:161‐167. 10.1007/978-3-319-16104-4_15 29787039

[54] Sakaguchi S , Yamaguchi T , Nomura T , Ono M . Regulatory T cells and immune tolerance. Cell. 2008;133(5):775‐787. 18510923 10.1016/j.cell.2008.05.009

[55] Crotty S . Follicular helper CD4 T cells (TFH). Annu Rev Immunol. 2011;29:621‐663. 21314428 10.1146/annurev-immunol-031210-101400

[56] Marracci GH , McKeon GP , Marquardt WE , Winter RW , Riscoe MK , Bourdette DN . α lipoic acid inhibits human T‐cell migration: Implications for multiple sclerosis. J Neurosci Res. 2004;78(3):362‐370. 15389837 10.1002/jnr.20255

[57] Salinthone S , Yadav V , Schillace RV , Bourdette DN , Carr DW . Lipoic acid attenuates inflammation via cAMP and protein kinase A signaling. PLoS One. 2010;5(9):e13058. 20927401 10.1371/journal.pone.0013058 PMC2946928

[58] Lee HA , Hughes DA . Alpha‐lipoic acid modulates NF‐kappaB activity in human monocytic cells by direct interaction with DNA. Exp Gerontol. 2002;37(2‐3):401‐410. 11772527 10.1016/s0531-5565(01)00207-8

[59] George JD , Kim E , Spain R , Bourdette D , Salinthone S . Effects of lipoic acid on migration of human B cells and monocyte‐enriched peripheral blood mononuclear cells in relapsing remitting multiple sclerosis. J Neuroimmunol. 2018;315:24‐27. 29306401 10.1016/j.jneuroim.2017.12.009

[60] Salinthone S , Schillace RV , Marracci GH , Bourdette DN , Carr DW . Lipoic acid stimulates cAMP production via the EP2 and EP4 prostanoid receptors and inhibits IFN gamma synthesis and cellular cytotoxicity in NK cells. J Neuroimmunol. 2008;199:46‐55. 18562016 10.1016/j.jneuroim.2008.05.003 PMC2561179

[61] Fiedler SE , Spain RI , Kim E , Salinthone S . Lipoic acid modulates inflammatory responses of monocytes and monocyte‐derived macrophages from healthy and relapsing‐remitting multiple sclerosis patients. Immunol Cell Biol. 2021;99(1):107‐115. 32762092 10.1111/imcb.12392

[62] Schillace RV , Pisenti N , Pattamanuch N , et al. Lipoic acid stimulates cAMP production in T lymphocytes and NK cells. Biochem Biophys Res Comm. 2007;354(1):259‐264. 17210133 10.1016/j.bbrc.2006.12.195 PMC4278348

[63] Chaudhary P , Marracci G , Pocius E , Galipeau D , Morris B , Bourdette D . Effects of lipoic acid on primary murine microglial cells. J Neuroimmunol. 2019;334:576972. 31176014 10.1016/j.jneuroim.2019.576972 PMC6660368

[64] Barsukova A , Forte M , Bourdette D . Lipoic acid prevents axonal spheroid formation and severing in adult neurons during oxidative stress. Multiple Sclerosis. 2012;18(4):213. 10.1177/1352458512459019

[65] Berer K , Mues M , Koutrolos M , et al. Commensal microbiota and myelin autoantigen cooperate to trigger autoimmune demyelination. Nature. 2011;479(7374):538‐541. 22031325 10.1038/nature10554

[66] Greer JM , Trifilieff E , Pender MP . Correlation between anti‐myelin proteolipid protein (PLP) antibodies and disease severity in multiple sclerosis patients with PLP response‐permissive HLA types. Front Immunol. 2020;11:1891. 32973782 10.3389/fimmu.2020.01891 PMC7473150

[67] Acs P , Kipp M , Norkute A , et al. 17beta‐estradiol and progesterone prevent cuprizone provoked demyelination of corpus callosum in male mice. Glia. 2009;57(8):807‐814. 19031445 10.1002/glia.20806

[68] Steelman AJ , Thompson JP , Li J . Demyelination and remyelination in anatomically distinct regions of the corpus callosum following cuprizone intoxication. Neurosci Res. 2012;72(1):32‐42. 22015947 10.1016/j.neures.2011.10.002 PMC3230728

[69] Marracci GH , Jones RE , McKeon GP , Bourdette DN . Alpha lipoic acid inhibits T cell migration into the spinal cord and suppresses and treats experimental autoimmune encephalomyelitis. J Neuroimmunol. 2002;131(1‐2):104‐114. 12458042 10.1016/s0165-5728(02)00269-2

[70] Morini M , Roccatagliata L , Dell'Eva R , et al. α‐Lipoic acid is effective in prevention and treatment of experimental autoimmune encephalomyelitis. J Neuroimmunol. 2004;148(1‐2):146‐153. 14975595 10.1016/j.jneuroim.2003.11.021

[71] Schreibelt G , Musters RJP , Reijerkerk A , et al. Lipoic acid affects cellular migration into the central nervous system and stabilizes blood‐brain barrier integrity. J Immunol. 2006;177(4):2630‐2637. 16888025 10.4049/jimmunol.177.4.2630

[72] Wang KC , Tsai CP , Lee CL , et al. α‐Lipoic acid enhances endogenous peroxisome‐proliferator‐activated receptor‐γ to ameliorate experimental autoimmune encephalomyelitis in mice. Clin Sci. 2013;125(7):329‐340. 10.1042/CS20120560 23550596

[73] Li B , Tan GJ , Lin HQ , Zhang JN , Guo L , Chen LP . Neuroprotective effects of α‐lipoic acid on long‐term experimental autoimmune encephalomyelitis. Eur Rev Med Pharmacol Sci. 2018;22(19):6517‐6528. 30338822 10.26355/eurrev_201810_16066

[74] Dietrich M , Helling N , Hilla A , et al. Early alpha‐lipoic acid therapy protects from degeneration of the inner retinal layers and vision loss in an experimental autoimmune encephalomyelitis‐optic neuritis model. J Neuroinflammation. 2018;15(1):71. 29514678 10.1186/s12974-018-1111-y PMC5840773

[75] Khan N , Gordon R , Woodruff TM , Smith MT . Antiallodynic effects of alpha lipoic acid in an optimized RR‐EAE mouse model of MS‐neuropathic pain are accompanied by attenuation of upregulated BDNF‐TrkB‐ERK signaling in the dorsal horn of the spinal cord. Pharmacol Res Perspect. 2015;3(3):e00137. 26171221 10.1002/prp2.137 PMC4492753

[76] Vesterinen HM , Connick P , Irvine CMJ , et al. Drug repurposing: a systematic approach to evaluate candidate oral neuroprotective interventions for secondary progressive multiple sclerosis. PLoS One. 2015;10(4):e0117705. 25856304 10.1371/journal.pone.0117705 PMC4391783

[77] Venugopal R , Jaiswal AK . Nrf2 and Nrf1 in association with Jun proteins regulate antioxidant response element‐mediated expression and coordinated induction of genes encoding detoxifying enzymes. Oncogene. 1998;17(24):3145‐3156. 9872330 10.1038/sj.onc.1202237

[78] Steele ML , Fuller S , Patel M , Kersaitis C , Ooi L , Münch G . Effect of Nrf2 activators on release of glutathione, cysteinylglycine and homocysteine by human U373 astroglial cells. Redox Biol. 2013;1(1):441‐445. 24191238 10.1016/j.redox.2013.08.006 PMC3814960

[79] Cheng Y , Luo F , Zhang Q , et al. α‐Lipoic acid alleviates pentetrazol‐induced neurological deficits and behavioral dysfunction in rats with seizures via an Nrf2 pathway. RSC Advances. 2018;8(8):4084‐4092.

[80] Xia D , Zhai X , Wang H , Chen Z , Fu C , Zhu M . Alpha lipoic acid inhibits oxidative stress‐induced apoptosis by modulating of Nrf2 signalling pathway after traumatic brain injury. J Cell Mol Med. 2019;23(6):4088‐4096. 30989783 10.1111/jcmm.14296 PMC6533507

[81] Chaudhary P , Marracci GH , Bourdette DN . Lipoic acid inhibits expression of ICAM‐1 and VCAM‐1 by CNS endothelial cells and T cell migration into the spinal cord in experimental autoimmune encephalomyelitis. J Neuroimmunol. 2006;175(1‐2):87‐96. 16644024 10.1016/j.jneuroim.2006.03.007

[82] Chaudhary P , Marracci G , Galipeau D , Pocius E , Morris B , Bourdette D . Lipoic acid reduces inflammation in a mouse focal cortical experimental autoimmune encephalomyelitis model. J Neuroimmunol. 2015;289:68‐74. 26616873 10.1016/j.jneuroim.2015.10.011 PMC4664888

[83] Edling AE , Choksi S , Huang Z , Korngold R . An organic CD4 inhibitor reduces the clinical and pathological symptoms of acute experimental allergic encephalomyelitis. J Autoimmun. 2002;18(2):169‐179. 11908949 10.1006/jaut.2001.0576

[84] Lawrence T . The nuclear factor NF‐kappaB pathway in inflammation. Cold Spring Harb Perspect Biol. 2009;1(6):a001651. 20457564 10.1101/cshperspect.a001651 PMC2882124

[85] Kim HS , Kim HJ , Park KG , et al. α‐Lipoic acid inhibits matrix metalloproteinase‐9 expression by inhibiting NF‐κB transcriptional activity. Exp Mol Med. 2007;39(1):106‐113. 10.1038/emm.2007.12 17334234

[86] Kobayashi EH , Suzuki T , Funayama R , et al. Nrf2 suppresses macrophage inflammatory response by blocking proinflammatory cytokine transcription. Nat Commun. 2016;7:11624. 27211851 10.1038/ncomms11624 PMC4879264

[87] Quinti L , Dayalan Naidu S , Träger U , et al. KEAP1‐Modifying Small Molecule Reveals Muted NRF2 Signaling Responses in Neural Stem Cells from Huntington's Disease Patients. Proceedings of the National Academy of Sciences; 2017;114(23):E4676. 10.1073/pnas.1614943114 PMC5468652 28533375

[88] Bernardo A , Minghetti L . Regulation of glial cell functions by PPAR‐gamma natural and synthetic agonists. PPAR Res. 2008;2008:864140. 18464925 10.1155/2008/864140 PMC2367430

[89] Villapol S . Roles of peroxisome proliferator‐activated receptor gamma on brain and peripheral inflammation. Cell Mol Neurobiol. 2018;38(1):121‐132. 28975471 10.1007/s10571-017-0554-5 PMC5776063

[90] Eslami H , Sharifi AM , Rahimi H , Rahati M . Protective effect of telmisartan against oxidative damage induced by high glucose in neuronal PC12 cell. Neurosci Lett. 2014;558:31‐36. 24211690 10.1016/j.neulet.2013.10.057

[91] Jung TW , Lee JY , Shim WS , et al. Rosiglitazone protects human neuroblastoma SH‐SY5Y cells against acetaldehyde‐induced cytotoxicity. Biochem Biophys Res Comm. 2006;340(1):221‐227. 16360119 10.1016/j.bbrc.2005.11.177

[92] Valizadeh A , Moassefi M , Barati E , Ali Sahraian M , Aghajani F , Fattahi M‐R . Correlation between the clinical disability and T1 hypointense lesions’ volume in cerebral magnetic resonance imaging of multiple sclerosis patients: a systematic review and meta‐analysis. CNS Neurosci Ther. 2021;27(11):1268‐1280. 34605190 10.1111/cns.13734 PMC8504532

[93] Sa MJ , de Sa J , Sousa L . Relapsing‐remitting multiple sclerosis: patterns of response to disease‐modifying therapies and associated factors: a national survey. Neurol Ther. 2014;3(2):89‐99. 26000225 10.1007/s40120-014-0019-4 PMC4386429

[94] Bilińska M , Frydecka I , Podemski R . Clinical course and changes of soluble interleukin‐2 receptor and soluble forms of intercellular adhesion molecule‐1 (ICAM‐1) in serum of multiple sclerosis patients. Neurol Neurochir Pol. 2001;35(1):47‐56. 11464716

[95] Loy BD , Fling BW , Horak FB , Bourdette DN , Spain RI . Effects of lipoic acid on walking performance, gait, and balance in secondary progressive multiple sclerosis. Complemen Ther Med. 2018;41:169‐174. 10.1016/j.ctim.2018.09.006 PMC6263172 30477834

[96] Khallli M , Eskandari G , Ghajarzadeh M , et al. Lipoic acid and multiple sclerosis: a randomized controlled conical trial. CurrTop Nutraceutical Res. 2012;10(2):95‐100.

[97] Lipoic Acid for Progressive Multiple Sclerosis (MS) . https://clinicaltrialsgov/ct2/results?pg=1&load=cart&id=NCT03161028.

[98] Cameron M , Taylor C , Lapidus J , Ramsey K , Koop D , Spain R . Gastrointestinal tolerability and absorption of R‐ versus R, S‐lipoic acid in progressive multiple sclerosis: a randomized crossover trial. J Clin Pharmacol. 2020;60(8):1099‐1106. 32212340 10.1002/jcph.1605

[99] Ross AP . Tolerability, adherence, and patient outcomes. Neurology. 2008;71(24 suppl. 3):S21‐S23. 19064871 10.1212/WNL.0b013e31818f3dcb

[100] Yadav V , Marracci G , Lovera J , et al. Lipoic acid in multiple sclerosis: a pilot study. Mult Scler. 2005;11(2):159‐165. 15794388 10.1191/1352458505ms1143oa

[101] Falardeau J , Fryman A , Wanchu R , et al. Oral lipoic acid as a treatment for acute optic neuritis: a blinded, placebo controlled randomized trial. Mult Scler J Exp Transl Clin. 2019;5(2):2055217319850193. 31205740 10.1177/2055217319850193 PMC6537072

[102] Vidovic B , Milovanovic S , Stefanovic A , et al. Effects of alpha‐lipoic acid supplementation on plasma adiponectin levels and some metabolic risk factors in patients with schizophrenia. J Med Food. 2017;20(1):79‐85. 10.1089/jmf.2016.0070 28009525

[103] Agathos E , Tentolouris A , Eleftheriadou I , et al. Effect of alpha‐lipoic acid on symptoms and quality of life in patients with painful diabetic neuropathy. J Int Med Res. 2018;46(5):1779‐1790. 29517942 10.1177/0300060518756540 PMC5991249

[104] Gatti M , Ippoliti I , Poluzzi E , et al. Assessment of adverse reactions to α‐lipoic acid containing dietary supplements through spontaneous reporting systems. Clin Nutr. 2020;40(3):1176‐1185. 32778460 10.1016/j.clnu.2020.07.028

[105] Fogacci F , Rizzo M , Krogager C , et al. Safety evaluation of alpha‐lipoic acid supplementation: a systematic review and meta‐analysis of randomized placebo‐controlled clinical studies. Antioxidants. 2020;9(10):1011. 10.3390/antiox9101011 PMC7603186 33086555

[106] Khalili M , Eghtesadi S , Mirshafiey A , et al. Effect of lipoic acid consumption on oxidative stress among multiple sclerosis patients: a randomized controlled clinical trial. Nutr Neurosci. 2014;17(1):16‐20. 23485514 10.1179/1476830513Y.0000000060

[107] Waslo CS , Chan BK , Taylor CA , Bourdette D , Linesman D , Spain RI . Evaluation of serum glutathione changes as a mechanism of action of lipoic acid. Mult Scler J. 2019;25:125.

[108] Seifar F , Khalili M , Azimi A . Effect of lipoic acid on oxidative stress in multiple sclerosis patients: a double blind randomised clinical trial. Eur J Neurol. 2016;23:825.

[109] Behzadi AH , Gupta A , Prince MR . Potential role of lipoic acid as a chelator in prevention and treatment of gadolinium brain retention. Med Hypotheses. 2018;114:29. 29602459 10.1016/j.mehy.2018.02.021

[110] Liuzzi GM , Trojano M , Fanelli M , et al. Intrathecal synthesis of matrix metalloproteinase‐9 in patients with multiple sclerosis: implication for pathogenesis. Mult Scler. 2002;8(3):222‐228. 12120694 10.1191/1352458502ms800oa

[111] Etienne‐Manneville S , Manneville JB , Adamson P , Wilbourn B , Greenwood J , Couraud PO . ICAM‐1‐coupled cytoskeletal rearrangements and transendothelial lymphocyte migration involve intracellular calcium signaling in brain endothelial cell lines. J immunol. 2000;165(6):3375‐3383. 10975856 10.4049/jimmunol.165.6.3375

[112] Durieu‐Trautmann O , Chaverot N , Cazaubon S , Strosberg AD , Couraud PO . Intercellular adhesion molecule 1 activation induces tyrosine phosphorylation of the cytoskeleton‐associated protein cortactin in brain microvessel endothelial cells. J Biol Chem. 1994;269(17):12536‐12540. 7909803

[113] Khoury SJ , Orav EJ , Guttmann CRG , Kikinis R , Jolesz FA , Weiner Hl . Changes in serum levels of ICAM and TNF‐R correlate with disease activity in multiple sclerosis. Neurology. 1999;53(4):758‐764. 10489037 10.1212/wnl.53.4.758

[114] Giovannoni G , Lai M , Thorpe J , et al. Longitudinal study of soluble adhesion molecules in multiple sclerosis: correlation with gadolinium enhanced magnetic resonance imaging. Neurology. 1997;48(6):1557‐1565. 9191766 10.1212/wnl.48.6.1557

[115] Waubant E , Goodkin DE , Gee L , et al. Serum MMP‐9 and TIMP‐1 levels are related to MRI activity in relapsing multiple sclerosis. Neurology. 1999;53(7):1397. 10534241 10.1212/wnl.53.7.1397

[116] Zhang Y , Lu W , Wang Z , et al. Reduced neuronal cAMP in the nucleus accumbens damages blood‐brain barrier integrity and promotes stress vulnerability. Biol Psychiat. 2020;87(6):526‐537. 31812254 10.1016/j.biopsych.2019.09.027

